# The Impact of Inflammatory Bowel Disease Clinic On-site Vaccination Services

**DOI:** 10.1093/crocol/otab067

**Published:** 2021-09-20

**Authors:** Nadeen Hussain, Deborah Proctor, Badr Al-Bawardy

**Affiliations:** 1 Department of Internal Medicine, Yale School of Medicine, New Haven, Connecticut, USA; 2 Section of Digestive Diseases, Department of Internal Medicine, Yale School of Medicine, New Haven, Connecticut, USA

**Keywords:** inflammatory bowel disease, vaccines, health maintenance

## Abstract

**Background:**

Despite being susceptible to vaccine-preventable diseases, patients with inflammatory bowel disease (IBD) have low vaccination rates. The aims of this study are to examine the rates of vaccine discussion and completion among patients of an IBD clinic that offers on-site vaccinations.

**Methods:**

This is a retrospective study from March 1, 2019 to February 1, 2020 comparing vaccination discussion and completion rates for patients with IBD who visited 2 clinics—1 that offers on-site vaccination (Clinic A) and 1 that does not (Clinic B). Both clinics are staffed by the same IBD physicians and utilize an identical IBD vaccine checklist.

**Results:**

A total of 356 patients were included (64.6% Crohn’s, 31.7% ulcerative colitis, 1.1% indeterminate colitis, and 2.5% pouchitis). Overall vaccine discussion rate was 77.6% in Clinic A vs 70.9% in Clinic B (*P* = .15). Herpes zoster (HZ), pneumococcal, and tetanus–diphtheria–pertussis (Tdap) vaccine discussion rates were higher in Clinic A compared to Clinic B (17.8% vs 5%, *P* < .001, 56.3% vs 43.4%; *P* = .01, and 41.4% vs 21.4%, *P* < .001), respectively. Influenza vaccine completion and hepatitis A immunization rates were higher in Clinic A compared to Clinic B (67.8% vs 47.8%, *P* < .001 and 36.2% vs 22.5%, *P* = .005), respectively. A numerically higher percentage of patients completed the pneumococcal, HZ, and hepatitis B vaccination in Clinic A, but this difference did not reach statistical significance.

**Conclusions:**

IBD clinic on-site vaccination services enhanced vaccine discussion and completion rates. IBD clinics should offer on-site vaccination services as part of the comprehensive care of the IBD patient.

## Introduction

Patients with inflammatory bowel disease (IBD) including Crohn’s disease and ulcerative colitis are susceptible to vaccine-preventable diseases.^1^ Susceptibility to these diseases is mainly related to immunosuppressive therapy, but other factors including inherent immune dysregulation, malnutrition, and comorbidities have also been implicated.^2^

Despite this increased risk, vaccination rates among patients with IBD remain low compared to the general population. A recent study of 239 IBD patients found varying rates of immunization: 72.4% for inactivated influenza, 48.3% for hepatitis B, and as low as 2.1% for herpes zoster (HZ).^3^ Another study of 195 IBD patients showed that 55% received hepatitis B vaccine, 7% received hepatitis A, and only 2% received pneumococcal vaccination.^4^

Multiple factors have been implicated in the suboptimal rates of vaccination discussion and completion in patients with IBD. These include lack of knowledge by primary care physicians and general gastroenterologist about recommended vaccinations of patients with IBD. According to a survey study of 276 primary care physicians who had experience with IBD patients, only 49% of providers frequently took immunization histories, and only 2.5% correctly recommended vaccinations all the time.^5^ Providers were more likely to recommend vaccinations to immunocompetent hosts, and up to 23% of primary care physicians incorrectly recommended live vaccines to immunocompromised hosts. Another study showed that only 52% of gastroenterologists asked their patients about their immunization history most or all of the time, and 20%–30% of gastroenterologists incorrectly recommended administering live vaccines to their immunosuppressed patients.^6^

Another important factor that has been associated with suboptimal vaccination rates in IBD is delegation of the responsibility to discuss and administer vaccines between primary care physicians and gastroenterologists. In a survey of 108 gastroenterologists, 64% thought that the primary care physician was responsible for discussing vaccines, and 83% thought that the primary care physician is responsible for administering vaccines in IBD patients.^6^ On the contrary, a survey of primary care physicians found only 37% felt comfortable providing preventative health services including vaccines for IBD patients. It is proposed that gastroenterologists should take the lead at educating and providing specific vaccine recommendations while primary care physicians assume the role of administering the vaccines.^7^

Previous studies have attempted to identify strategies to improve vaccination such as patient education and using standardized checklists. One study showed that there was a significant increase in vaccine uptake for patients who received disease-specific information through the electronic health record portal.^8^ Another study demonstrated that self-reporting of vaccination status, particularly influenza vaccination status, is an effective way to determine vaccination status and provides useful information for receipt of vaccines for patients with IBD.^9^ One practical strategy is providing vaccinations to patients directly in the IBD clinic. Experts have previously outlined steps to implement a successful vaccine program in outpatient gastroenterology clinics, including identifying a vaccine champion, collaborating with the pharmacy department, and training appropriate staff.^10^ The purpose of this study is to examine the vaccination completion rates among patients of IBD clinics that offer vaccinations on-site vs patients of IBD clinics that do not offer vaccinations on-site and are instead referred to their primary care physician or pharmacy for administration of vaccines.

## Materials and Methods

This study was approved by the Yale University Institutional Review Board (IRB#2000027302).

This study is a retrospective chart review from March 1, 2019 to February 1, 2020 of all patients with IBD who attended IBD clinics at Yale University School of Medicine: Clinic A, which offers on-site vaccination services, and Clinic B, which does not. Inclusion criteria were: age ≥18 years, diagnosis of IBD (Crohn’s disease, ulcerative colitis, indeterminate colitis, and pouchitis), and at least 2 consecutive clinic visits during the study period. Exclusion criteria were: age <18, non-IBD diagnosis, and pregnancy. Both Clinic A and Clinic B are staffed by same 2 dedicated IBD physicians, and the same IBD nurse. Both Clinic A and Clinic B were regularly attended by gastroenterology fellows, had identical templated schedules and are outpatient clinics and not hospital-based clinics. An electronic medical record preventative health services IBD checklist is available and utilized at both clinic locations. In clinic A, the IBD nurse administered vaccines to patients on-site immediately after conclusion of the clinical visit. Vaccines were revenue generating for the clinic. In clinic B, patients were referred to their primary care physicians or local pharmacy for vaccine administration. All listed vaccines were available except human papilloma virus (HPV) and HZ (temporarily unavailable due to shortages) vaccines.

The following demographic variables were collected from the electronic medical record for each patient: clinic location (Clinic A and Clinic B), sex, age, body mass index, IBD subtype (Crohn’s disease, ulcerative colitis, indeterminate colitis, or pouchitis), disease location, phenotype, and duration. Medical therapy details including type of agent used such as mesalamine/5-ASA, biologic/small molecule therapy, steroid therapy, immunomodulator monotherapy (methotrexate or thiopurine), combination biologic and immunomodulator therapy were collected. Other pertinent factors such as prior bowel resection, smoking, clinical disease activity, and endoscopic disease activity were abstracted.

Data were then collected by chart review of the clinical notes that documented whether or not discussion was had about vaccines in general, and also specifically for the influenza, pneumonia, HZ, hepatitis A, hepatitis B, tetanus–diphtheria–pertussis (Tdap), and HPV vaccines. Patients were considered eligible for the pneumococcal vaccine if they were on immunosuppressive therapy as recommended by the American College of Gastroenterology guidelines.^7^ Patients were considered eligible for the HZ vaccines if they were over the age of 50 or initiating/on tofacitinib therapy. Patients were considered candidates for the HPV vaccination if they were between the ages of 19–26, which was the Advisory Committee on Immunization Practices (ACIP) recommendation at the time of the study.^11^

In addition, data were collected on whether or not the aforementioned vaccines were administered or up to date. For hepatitis A and B, positive serology served as a marker of prior immunization. In order to find these data, the immunization tab in the chart was reviewed. This tab listed vaccines administered through our health system, and also provided a link to immunizations administered outside of the health system (ie, pharmacies). The outcomes of the study are: (1) differences in overall and specific vaccination discussion rates (defined as documented vaccine discussion in the clinical note) between Clinic A and Clinic B and (2) differences in specific vaccination completion rates between Clinic A and Clinic B.

JMP (SAS Institute Inc.) statistical software was used for data analysis. Proportions were presented with descriptive statistics. Student *t*-test and Pearson’s chi-square test were applied to continuous and categorical data, respectively. A *P* value <.05 was considered statistically significant.

## Results

A total of 356 patients were included in the study. The median age was 41 (range: 19–95) and 51.7% of patients were female. With respect to IBD subtype, 64.6% of patients had Crohn’s disease, 31.7% had ulcerative colitis, 1.1% had indeterminate colitis, and 2.5% had pouchitis (Table 1).

**Table 1.** Baseline characteristics

	Value
Median age, years (range)	41 (19–95)
Female, *n* (%)	184 (51.7)
BMI, median (range)	26 (14–53)
Disease duration, median (range)	13 (1–60)
IBD subtype	
Crohn’s disease, *n* (%)	230 (64.6)
L1	36(15.7%)
L2	48 (20.9%)
L3	146 (63.4%)
L4	0
B1	90 (39.1%)
B2	52 (22.6%)
B3	88 (38.3%)
Perianal disease, *n* (%)	70 (19.7)
Ulcerative colitis, *n* (%)	113 (31.7)
Indeterminate colitis, *n* (%)^a^	4 (1.1)
Proctitis	6 (5.1%)
Proctosigmoiditis	12 (10.3%)
Left-sided colitis	24 (20.5%)
Pancolitis	74 (63.2%)
Pouchitis	9 (2.5)
Tobacco use, *n* (%)	31 (8.7)
Prior bowel resection, *n* (%)	123 (34.6)

Abbreviations: BMI, body mass index; IBD, inflammatory bowel disease.

^a^One patient with indeterminate colitis did not have documented disease extent.

In terms of medical therapy, 64% of patients were on biologic/small molecule therapy, with an additional 16.7% of patients on combination therapy with a biologic and an immunomodulator (Table 2). Corticosteroid use was noted in 10.1% (*n* = 36). The breakdown of the different therapeutic specific agents is illustrated in Table 2. Within the whole cohort, at least 1 vaccine was discussed with 74.2% of patients (*n* = 264).

**Table 2.** Medical management

	Value
Biologic/small molecule, *n* (%)	227 (63.8)
Infliximab	80 (35.2%)
Adalimumab	60 (26.4%)
Ustekinumab	49 (21.6%)
Vedolizumab	33 (14.5%)
Tofacitinib	3 (1.3%)
Combination biologics	2 (1.0%)
Immunomodulator monotherapy, *n* (%)	26 (7%)
Thiopurine	23 (88.5%)
Methotrexate	3 (11.5%)
Combination biologic and immunomodulator, *n* (%)	59 (16.7%)
Corticosteroids (prednisone and budesonide), *n* (%)	36 (10.1%)
Mesalamine, *n* (%)	76 (21.3%)

A total of 174 patients were in the Clinic A group while 182 patients were in the Clinic B group. There was no significant difference between the 2 groups in terms of age, sex, race, IBD subtype, duration of IBD, and insurance type (Table 3). Biologic monotherapy was used in 68% of patients in Clinic A vs 59% of patients in Clinic B (*P* = .08). Immunomodulator monotherapy was utilized in 6.4% and 8.2% in Clinic A and Clinic B patients, respectively (*P* = .50). Combination biologic and immunomodulator therapy was observed in 20.1% in Clinic A patients compared to 13.2% in Clinic B patients (*P* = .08). Any immunosuppression (defined as either biologic, small molecule, or immunomodulator therapy) was noted in 130 (74.7%) in Clinic A and 123 (67.6%) of patients in Clinic B (*P* = .14).

**Table 3.** Comparison of on-site (Clinic A) and non-on-site (Clinic B) vaccination locations

Variable	Clinic A (*n* = 174)	Clinic B (*n* = 182)	P
Mean age, years (SD)	44.5 (15.4)	44.1 (15.5)	.78
Female, *n* (%)	92 (52.9%)	92 (50.6%)	.66
Race, *n* (%)			.11
White	136 (78.2%)	133 (73.1%)	
African American	11 (6.3%)	27 (14.8%)	
Hispanic	6 (3.4%)	4 (2.2%)	
Asian	17 (9.8%)	15 (8.2%)	
Pacific Islander	3 (1.7%)	2 (1%)	
American Indian	0 (0%)	1 (0.5%)	
Other/Not Listed	1 (0.6%)	0 (0%)	
Disease duration, mean (SD)	14.9 (11.1)	16.4 (11.8)	.21
IBD subtype, *n* (%)			.74
Crohn’s disease	112 (64.4%)	118 (64.8%)	
Ulcerative colitis	54 (31.0%)	59 (32.4%)	
Indeterminate colitis	2 (1.1%)	2 (1.1%)	
Pouchitis	6 (3.5%)	3 (1.7%)	
Biologic/small molecule, *n* (%)	119 (68.4%)	108 (59.3%)	.08
Immunomodulator monotherapy, *n* (%)	11 (6.4%)	15 (8.2%)	.50
Combination biologic and immunomodulator, *n* (%)	35 (20.1%)	24 (13.2%)	.08
Any immunosuppression, *n* (%)	130 (74.7%)	123 (67.6%)	.14
Insurance type, *n* (%)			.49
Private	125 (71.8%)	121 (66.5%)	
Medicare	16 (9.2%)	14 (7.7%)	
Medicaid	28 (16.1%)	41(22.5%)	
Self-pay	2 (1.1%)	4 (2.2%)	
Other	3 (1.7%)	2 (1.1%)	
Tobacco use, *n* (%)	18 (10.3%)	13 (7.1%)	.43

Abbreviation: IBD, inflammatory bowel disease.

**Figure 1.**
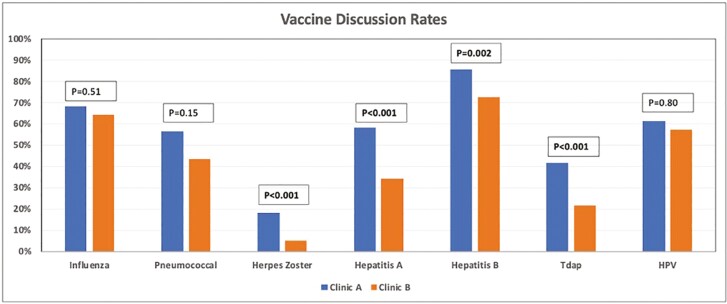
Bar graph showing the vaccination discussion rates between Clinic A and Clinic B. Abbreviations: HPV, human papilloma virus; Tdap, tetanus–diphtheria–pertussis.

Vaccines were discussed in Clinic A with 77.6% of the patients and with 70.9% of the patients in Clinic B (*P* = .15) (Figure 1). The influenza vaccine discussion rate was 67.8% for Clinic A and 64.3% for Clinic B (*P* = .51). The pneumococcal vaccine discussion rate was 56.3% in Clinic A and 43.4% in Clinic B (*P* = .15). A total of 132 patients met the eligibility criteria for the HZ vaccine. Out of these patients, the HZ vaccine discussion rate was 17.8% in Clinic A and 5% in Clinic B (*P* < .001). In Clinic A, 58.1% of patients had hepatitis A serologies checked compared to 34% in Clinic B (*P* < .001). In Clinic A, 85.6% of patients had hepatitis B serologies checked compared to 72.5% in Clinic B (*P* = .002). The Tdap discussion rate was 41.4% in Clinic A compared to 21.4% in Clinic B (*P* < .001). A total of 44 patients met the eligibility criteria for the HPV vaccine. HPV vaccination discussion rates were 60.9% in the Clinic A group compared to 57.1% in the Clinic B group (*P* = .80).

**Figure 2.**
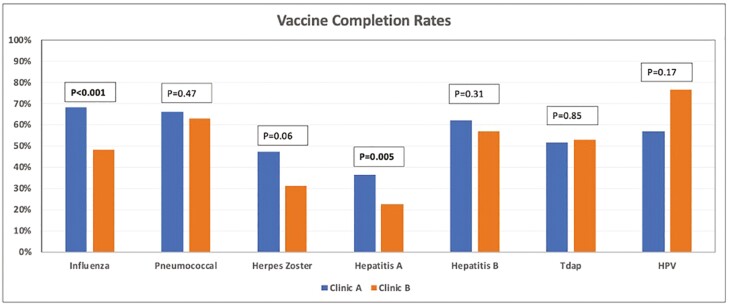
Bar graph showing the vaccination completion rates between Clinic A and Clinic B. Abbreviations: HPV, human papilloma virus; Tdap, tetanus–diphtheria–pertussis.

The influenza vaccine completion rate was 67.8% in Clinic A and 47.8% in clinic B (*P* < .001) (Figure 2). The pneumococcal vaccine completion rate was 65.9% in Clinic A and 62.6% in Clinic B (*P* = .47). The HZ vaccine completion rate was 47.1% in Clinic A and 31.1% in Clinic B (*P* = .06). Immunity to hepatitis A was noted in 36.2% of patients in Clinic A compared to 22.5% in Clinic B (*P* = .005). Documentation of immunity or receipt of the hepatitis B vaccine was noted in 62.1% of patients in Clinic A compared to 56.7% in Clinic B (*P* = .31). Tdap completion rate was 51.7% in Clinic A compared to 52.8% in Clinic B (*P* = .85). HPV vaccination completion rates were 56.5% in the Clinic A group compared to 76.2% in the Clinic B group (*P* = .17).

## Discussion

Our study compared vaccination discussion and completion rates in a cohort of patients with IBD who attended an IBD clinic which offered on-site vaccination services (Clinic A) vs an IBD clinic that did not (Clinic B). We found the rate of overall discussion of vaccines was similar between the 2 groups. Certain vaccines, however, were discussed at higher rate in Clinic A including pneumococcal, HZ, hepatitis A, hepatitis B, and Tdap. In terms of vaccine completion, influenza and hepatitis A vaccine completion rates were significantly higher in the Clinic A group. In addition, pneumococcal, HZ, and hepatitis B completion rates were numerically higher in the Clinic A group compared to Clinic B. We believe that the convenience and immediate availability of vaccines enhances vaccine completion rate.

Patients with IBD are at risk for acquiring vaccine-preventable diseases, particularly related to immunosuppressive therapy. Despite the increased risk, the rates of vaccinations of patients with IBD have been reported to be low. A more recent large immunization survey study showed an improvement in the rates of preventative services offered to IBD patients, but the absolute rate of vaccination completion remains low (48.4% for the annual influenza vaccine).^12^ Our study showed an overall rate of vaccination discussion of 74.2%.

Multiple factors have been identified as barriers to vaccination completion in patients with IBD. These factors include lack of knowledge by patient and provider, lack of discussion between patient and provider, and concern about vaccine safety/vaccine hesitancy.^13^ Previous studies have attempted to identify strategies to overcome these barriers. One study of 505 patients randomized subjects into 2 groups, 1 that received standard clinical practice, and 1 that received additional education by a specialized IBD nurse including an immunization brochure and vaccination card. Eight months later, vaccination completion rates were higher in the intervention group compared to the control group (*P* < .001).^14^ However, the vaccination completion rate was only 33% in the intervention group. Another study administered a proforma to 30 gastroenterologists at 8 different IBD centers and evaluated vaccination rates before and afterwards. Introduction of the proforma increased self-reported gastroenterologist screening for vaccination history from 47% to 97% (*P* < .001). However, only 42%, 39%, 66%, and 49% of patients followed the recommendations and were vaccinated against hepatitis B, varicella, influenza, and pneumococcus, respectively.^15^ This emphasizes that discussion of vaccines does not necessarily translate into receiving and completing the recommended vaccinations, which is the ultimate goal of providing preventative health services in the IBD population.

One of the main barriers to vaccine completion is the role designation of vaccine administrator. Previous research has indicated that up to 2/3rd of gastroenterologists thought that the primary care physician was responsible for discussing vaccines.^6^ Moreover, up to 80% of gastroenterologists thought that the primary care physician was responsible for administering vaccines in IBD patients. On the contrary, only 1/3rd of primary care physicians felt comfortable providing preventative health services including vaccines for IBD patients.^6^ This highlights the need to clearly define the role of the gastroenterologist in providing preventative health services such as vaccines. Therefore, the American College of Gastroenterology released guidelines in 2017 encouraging gastroenterologists to take the lead in providing preventative health services including vaccines in patients with IBD.^7^ Our results demonstrate that by providing on-site vaccine administration services, we can improve the vaccines completion rates.

The strengths of our study include having a relatively large patient sample. We also provide a control group with similar baseline characteristics to the intervention group (Clinic A). Also, patients in Clinic A and Clinic B were evaluated by the same team of IBD physicians and IBD nurse using an identical electronic medical record checklist which limits confounding of our results. The limitations of this study include inherent limitations of a retrospective study design and relatively short follow-up period. Another limitation is that there may be socioeconomic differences between the populations at the 2 clinics, which may impact rates of vaccination that we could not capture with our current data. Based on these data, prospective studies should be pursued to ascertain the impact of on-site vaccination administrative services.

In conclusion, providing IBD clinic on-site vaccination services enhanced vaccine discussion and completion rates for certain vaccines. IBD clinics should consider offering on-site vaccination services as part of the comprehensive care of the IBD patient to improve vaccination completion rates.

## Data Availability

The raw data of this study are available upon request from the corresponding author.
